# Online Health-Searching Behavior Among HIV-Seropositive and HIV-Seronegative Men Who Have Sex With Men in the Baltimore and Washington, DC Area

**DOI:** 10.2196/jmir.2479

**Published:** 2013-05-03

**Authors:** Ying Li, J Polk, Michael Plankey

**Affiliations:** ^1^Division of Infectious DiseasesDepartment of MedicineGeorgetown University Medical CenterWashington, DCUnited States

**Keywords:** Internet, information seeking behavior, HIV infections, chronic disease, patient care

## Abstract

**Background:**

Searching online for health information is common among American adults. However, there have been few studies to investigate the online health-searching behaviors among men who have sex with men (MSM) with human immunodeficiency virus (HIV).

**Objective:**

To estimate the prevalence of Internet use among HIV-seropositive MSM and compare their online behaviors with HIV-seronegative men with chronic disease(s).

**Methods:**

This study was performed at the Baltimore/Washington, DC site of the Multicenter AIDS Cohort Study (MACS). A total of 200 MACS participants were asked to answer a self-administered questionnaire on a first-come basis during a semiannual study visit (from July to November 2011); 195 (97.5%) participants completed the survey. Multiple logistic regression models were used to investigate the factors influencing their online health-searching behaviors.

**Results:**

The median age of the 195 MSM participants was 57 years, 64.6% were white, 59.0% were employed, and 88.2% had Internet access at home and/or other locations. Of the 95 HIV-seropositive participants, 89.5% currently used highly active antiretroviral therapy (HAART) and 82.1% had Internet access. After adjusting for age and race/ethnicity, the HIV-seropositive participants were less likely to perform online searches for general disease-related information compared to the HIV-seronegative men with chronic disease(s) (OR 0.20, 95% CI 0.06-0.68, *P*=.01). There were no statistically significant associations with HIV status and searching for new medications/treatments (OR 0.55, 95% CI 0.19-1.55, *P*=.26) or support/advice from other patients (OR 0.52, 95% CI 0.18-1.53, *P*=.24). Increasing age by 5 years led to a decrease by 29% in the odds of online health-related searches for general information (OR 0.71, 95% CI 0.52-0.98, *P*=.03) and 26% for support/advice from other patients (OR 0.74, 95% CI 0.56-0.98, *P*=.03). A decrease of 25% for new medications/treatments was also seen, but was not statistically significant (OR 0.75, 95% CI 0.57-1.01, *P*=.06).

**Conclusions:**

This study shows that HIV-seropositive MSM have similar online health-searching behaviors as HIV-seronegative men with chronic disease(s). Independent of HIV status, older MSM are less likely to perform online health-related searches.

## Introduction

Approximately 80% of American adults used the Internet in 2010 [1]. For Internet users, searching online for health information has become highly prevalent. A national survey in 2005 found 59% of Internet users did online searching for health information for themselves [2]. A Pew Internet & American Life Project survey showed that 83% of Internet users searched for health information in 2010 [1]. Some studies have shown a high prevalence of health-related Internet use among people with human immunodeficiency virus (HIV) and suggested such Internet use to be beneficial in coping, support, and self-control in health behaviors [3-17]. Studies have also demonstrated that Internet use among people living with chronic diseases (such as diabetes and cancer) provides a sense of support and self-management [18-28].

A large number of studies [25,29-32], including a recent World Health Organization report [33], have suggested that HIV has shifted from a rapidly debilitating and fatal illness to a manageable chronic disease. However, few studies have compared the online health-searching behaviors of HIV-seropositive people with those of HIV-seronegative people with chronic disease(s).

We conducted a study of the online health-searching behaviors among the HIV-seropositive and HIV-seronegative men who have sex with men (MSM) currently enrolled in the Baltimore/Washington, DC site of the Multicenter AIDS Cohort Study (MACS). The aims of this study were to estimate the prevalence of Internet use and online heath-searching behaviors among the HIV-seropositive MSM compared to the HIV-seronegative men with chronic disease(s), and to identify the factors influencing these behaviors.

## Methods

### Recruitment

The MACS is an ongoing prospective study of the natural and treated histories of HIV infection among MSM in the United States. A total of 6972 men were recruited (4954 in 1984-1985, 668 in 1987-1991, and 1350 in 2001-2003) at 4 centers located in Baltimore/Washington, DC; Chicago; Los Angeles; and Pittsburgh. The study design of the MACS has been described in detail previously [34,35] and only methods relevant to the present study are presented here. All MACS questionnaires are available on the MACS website. MACS study protocols were approved by the institutional review boards of each of the participating centers, their community partners, and community advisory boards, and informed consent was obtained from all participants. MACS participants return every 6 months for detailed interviews, physical examinations, and collection of blood for laboratory testing and storage in a central repository. The interview includes a battery of questions related to medical conditions, medical treatments, sexual behavior, illicit drug use, and alcohol consumption since the previous visit. For this study, 200 participants (150 from Baltimore and 50 from Washington, DC) answered a self-administered questionnaire (see Multimedia Appendix 1 for details) on a first-come basis during their routine semiannual study visits from July to November 2011.

### Questionnaire

Participants were asked about HIV or chronic disease(s) (including cancer, diabetes, depression, kidney disease, erectile dysfunction, etc); about their Internet access and where they accessed the Internet (no access, only at home, only at other locations including workplace and library, or both at home and other locations); the number of hours per week they used the Internet for personal purposes (1-2, 3-4, 5-9, ≥10 hours/week); and about whether they have used online disease-related searches for general information, new medications/treatments, and support/advice from other patients.

The outcomes of interest were the hours of Internet use and online disease-related searching. Participants’ HIV status, age, and race/ethnicity (obtained from the centralized MACS database) were included as covariates.

### Statistical Analysis

Descriptive statistics were generated by HIV status. The prevalence of Internet access was calculated. Univariate associations of Internet access with participant’s employment status, race/ethnicity, age, and HIV status were examined using chi-square tests or Fisher exact tests for categorical variables, or Mann-Whitney tests for continuous variables. The associations between online behaviors and HIV status were investigated by using multiple logistic regression models. Age and race/ethnicity were included in the model. Statistical significance was evaluated at the .05 level. All analyses were performed using SAS version 9.2 (SAS Institute, Inc, Cary, NC, USA).

## Results

Demographics, health conditions, and Internet access of the participants in this study are shown in Table 1. The median age was 57 years (IQR 51-63). Most were white (64.6%) and employed (59.0%), including full-time, part-time, and self-employment. Of the 195 participants completing the questionnaire, 172 (88.2%) used the Internet, 149 (76.4%) had Internet access at home, and 24 (12.3%) used a smartphone to access the Internet for general health. Of the 95 HIV-seropositive participants, 85 (89.5%) currently used highly active antiretroviral therapy (HAART) and 78 (82.1%) had Internet access. Of the 100 HIV-seronegative participants, 34 (34.0%) had cancer or other chronic disease(s). Having Internet access was statistically significantly associated with participant’s employment status (*P*=.046), race/ethnicity (*P*<.001), and HIV status (*P*=.004), but was not associated with participant’s age (*P*=.30). Internet access was higher among the employed participants compared to the unemployed (93.8% vs 84.9%), the white participants compared to the nonwhite participants (96.8% vs 76.5%), and the HIV-seronegative compared to the HIV-seropositive (95.9% vs 83.0%) (data not shown).

**Table 1:** Demographics, health condition, and Internet access of the participants in the study.

Variable		HIV serostatus		All participants N=195
		HIV– n=100	HIV+ n=95	
Age (years), median (IQR)		60 (53-65)	54 (49-60)	57 (51-63)
**Race, n (%)**				
	White	80 (80.0)	46 (48.4)	126 (64.6)
	Nonwhite	20 (20.0)	49 (51.6)	69 (35.4)
**Employment, n (%)**				
	Employed^a^	69 (69.0)	46 (48.4)	115 (59.0)
	Unemployed	30 (30.0)	43 (45.3)	73 (37.4)
	No response	1 (1.0)	6 (6.3)	7 (3.6)
**Current therapy use, n (%)**				
	No therapy use	—	6 (6.3)	—
	HAART	—	85 (89.5)	—
	Combination therapy	—	4 (4.2)	—
**Chronic disease (except HIV), n (%)**				
	None	45 (45.0)	—	—
	Cancer only	5 (5.0)	—	—
	Other chronic condition^b^	26 (26.0)	—	—
	Both cancer and other(s)	3 (3.0)	—	—
	No response	21 (21.0)	—	—
Self-reported HIV, n (%)		—	62 (65.3)	—
**Internet access, n (%)**				
	No	4 (4.0)	16 (16.8)	20 (10.3)
	Only at home	42 (42.0)	38 (40.0)	80 (41.0)
	Only at other locations^c^	10 (10.0)	13 (13.7)	23 (11.8)
	Both home and others	42 (42.0)	27 (28.4)	69 (35.4)
	No response	2 (2.0)	1 (1.1)	3 (1.5)
**Smartphone, n (%)**				
	Internet for general health	11 (11.0)	13 (13.7)	24 (12.3)

^a^including full-time, part-time, and self-employment

^b^Other chronic conditions included depression, kidney disease, erectile dysfunction, diabetes, arthritis, high blood pressure, heart disease, etc.

^c^Other locations included workplace and library.

The online behaviors of the HIV-seropositive participants were compared to the HIV-seronegative men with chronic disease(s) (see Table 2), of which 23 participants without Internet access (or no response), 61 HIV-seronegative participants without chronic disease(s) (or no response), and 17 HIV-seropositive participants who did not report their HIV infection in the survey were excluded. Of 61 HIV-seropositive participants and 33 HIV-seronegative participants with chronic disease(s), 21 (34.4%) and 12 (36.4%) spent ≥10 hours per week on the Internet for personal searching, respectively. There were no statistically significant differences between these 2 groups (OR 1.03, 95% CI 0.38-2.74, *P*=.96).

**Table 2:** Online behaviors of the HIV-seropositive participants compared to the HIV-seronegative participants with chronic disease.

Online behavior		HIV serostatus, n (%)	
		HIV– with chronic disease n=33	HIV+ n=61
**Personal Internet use (hours/week)**			
	1-2	5 (15.2)	14 (22.9)
	3-4	11 (33.2)	12 (19.7)
	5-9	5 (15.2)	14 (23.0)
	≥10	12 (36.4)	21 (34.4)
	No response	0	
**Online disease-related^a^ search for general information**			
	No	6 (18.2)	23 (37.7)
	Yes	25 (75.7)	37 (60.7)
	No response	2 (6.1)	1 (1.6)
**Online disease-related search for new medications or treatments**			
	No	10 (30.3)	22 (36.1)
	Yes	21 (63.6)	38 (62.3)
	No response	2 (6.1)	1 (1.6)
**Online disease-related search for support or advice from other patients**			
	No	18 (54.5)	33 (54.1)
	Yes	12 (36.4)	25 (41.0)
	No response	3 (9.1)	3 (4.9)

^a^HIV-related information for the HIV-seropositive participants and specific disease-related information for the HIV-seronegative men with chronic disease(s).

After adjusting for age and race/ethnicity, the HIV-seropositive participants were less likely to search online for general disease-related information compared to the HIV-seronegative men with chronic disease(s) (OR 0.20, 95% CI 0.06-0.68, *P*=.01). There were no statistically significant differences in searching for new medications/treatments (OR 0.55, 95% CI 0.19-1.55, *P*=.26) or for support/advice from other patients (OR 0.52, 95% CI 0.18-1.53, *P*=.24) between these 2 groups. Increasing age by 5 years led to a decrease by 29% in the odds of online health-related searches for general information (OR 0.71, 95% CI 0.52-0.98, *P*=.03) and 26% for support/advice from other patients (OR 0.74, 95% CI 0.56-0.98, *P*=.03). A decrease of 25% for new medications/treatments was also seen, but was not statistically significant (OR 0.75, 95% CI 0.57-1.01, *P*=.06).

**Table 3:** Analysis results of multiple logistic regressions.

Covariates	Internet use ≥10 hours/week		Online disease-related search					
			General information		New medications/ treatments		Support/advice	
	OR (95% CI)	*P*	OR (95% CI)	*P*	OR (95% CI)	*P*	OR (95% CI)	*P*
Age (5-yr increase)	0.99 (0.77, 1.27)	.95	0.71 (0.52, 0.98)	.03	0.75 (0.57, 1.01)	.06	0.74 (0.56, 0.98)	.03
Nonwhite vs white	0.70 (0.26, 1.92)	.49	1.30 (0.44, 3.83)	.64	1.07 (0.38, 3.01)	.90	2.66 (0.95, 7.39)	.06
HIV+ vs HIV with chronic disease	1.03 (0.38, 2.74)	.96	0.20 (0.06, 0.68)	.01	0.55 (0.19, 1.55)	.26	0.52 (0.18, 1.53)	.24

## Discussion

To the best of our knowledge, this study is the first to compare the online behaviors among HIV-seropositive and HIV-seronegative MSM with chronic disease(s). We found that the HIV-seropositive participants showed similar online health-searching behaviors compared to the HIV-seronegative with chronic disease(s) participants. Independent of HIV status, older participants were less likely to do online health-related research.

In this study, 88% of the surveyed participants had Internet access, close to the prevalence (79%) among American adults in a 2010 report from the Pew Research Center [1]. In our study, 82% of the HIV-seropositive participants had access to Internet, more than the reported 56% from earlier studies of people living with HIV and/or acquired immune deficiency syndrome (AIDS) [9,13]. Among our HIV-seropositive participants, 68% had Internet access at home, more than twice than the number (33%) reported by Kalichman et al in 2002 [13].

It has been known that HIV disease meets several chronic disease criteria, including an uncertain course, a prescribed treatment regimen, requirements of self-care and management, changes in roles and relationships, shifts in identity, and psychological distress [25,32]. Because the health care goal is to control symptoms and prevent disability rather than cure them [25], both HIV infection and chronic diseases require considerable patient self-care or self-monitoring of symptoms [32]. As such, it was not unexpected that our results showed no statistical differences in the online health-searching behaviors of HIV-seropositive participants and HIV-seronegative participants with chronic disease(s).

Coursaris and Liu [6] found that support-related information, including advice, referral, situation appraisal, and teaching, was exchanged most frequently in online HIV/AIDS self-help groups, which is in agreement with our observations. Of the HIV-seropositive participants with Internet access, 41% searched online for support/advice from other patients and 61% searched online for HIV-related information, more than the finding of a study of people living with HIV/AIDS that reported 45% for Internet health information-seeking behavior [36].

This study also showed that the odds of online health-related searching decreased with increasing age, in agreement with a recent report from the Pew Research Center that showed American adults older than 65 years were less likely than other age groups to use the Internet [37].

Certain limitations of this study deserve attention. We surveyed a convenience sample of MACS participants. Self-administration of the questionnaire did not provide an opportunity for participants to ask questions about the survey, so we could not verify the accuracy of the participants’ interpretation of the survey questions and, thus, their responses. In addition, our sample was limited to participants enrolled in the Baltimore/Washington, DC site; therefore, our results may not be generalizable to all MACS participants or all HIV-infected men. Recall bias may have been present.

Although the sample size in this study is small, our results provide valuable insight into the expanding Internet use of health self-management of HIV-seropositive MSM and HIV-seronegative MSM with chronic disease(s). Further study is needed to substantiate these findings. The high prevalence of online health searching observed among the MSM participants in the Baltimore/Washington, DC site will encourage MACS to expand the online health-searching behavior study to all sites.

## Multimedia Appendix 1

Questionnaire.

## Abbreviations

AIDS: acquired immune deficiency syndrome
HAART: highly active antiretroviral therapy
HIV: human immunodeficiency virus
MACS: Multicenter AIDS Cohort Study
MSM: men who have sex with men
