# Supplementary material for: Online Health-Searching Behavior Among HIV-Seropositive and HIV-Seronegative Men Who Have Sex With Men in the Baltimore and Washington, DC Area
Source: J Med Internet Res. 2013 May 3;15(5):e78. doi: 10.2196/jmir.2479 (PMC3650934; doi:10.2196/jmir.2479)
Supplement: Supplementary file 1 [file jmir_v15i5e78_app1.pdf]

Appendix 1. Questionnaire

Internet Access

Do you have access to the Internet? Please check all that apply.

[ ] No

[ ] At home

[ ] At work, the library or other location(s)

Smartphones

Do you use a cell phone to access the Internet for information about your general health?

[ ] Yes

[ ] No

The following questions only for the pariticipants with Internet Access

Internet: Hours Per Week

Since your last SHARE study visit, how many hours per week have you spent online for personal (non-work) matters?

[ ] 1-2 hours per week

[ ] 3-4 hours per week

[ ] 5-9 hours per week

[ ] 10 or more hours per week

Health Condition

You have been diagnosed with the following conditions:

HIV infection

[ ] Yes

[ ] No

Cancer

[ ] Yes

[ ] No

Do you have another health condition (not mentioned previously) that has impacted your life significantly? Examples might include, but are not limited to: depression, kidney disease, erectile dysfunction.

[ ] Yes

[ ] No

Please specify just one additional health condition (not mentioned in previous questions).

Type of Information Online

Since your last SHARE study visit, how often have you looked for these types of health condition-related information online?

General information about the condition

[ ] Frequently

[ ] Sometimes

[ ] Rarely

[ ] Never

[ ] Do Not Know

New medications or treatments

[ ] Frequently

[ ] Sometimes

[ ] Rarely

[ ] Never

[ ] Do Not Know

Support or advice from people in a similar health situation

[ ] Frequently

[ ] Sometimes

[ ] Rarely

[ ] Never

[ ] Do Not Know

Other

[ ] Frequently

[ ] Sometimes

[ ] Rarely

[ ] Never

[ ] Do Not Know

If you checked "Other", please specify.
